# Phylogenetic Diversity, Distribution, and Cophylogeny of Giant Bacteria (*Epulopiscium*) with their Surgeonfish Hosts in the Red Sea

**DOI:** 10.3389/fmicb.2016.00285

**Published:** 2016-03-14

**Authors:** Sou Miyake, David K. Ngugi, Ulrich Stingl

**Affiliations:** Marine Microbial Ecology, Red Sea Research Center, King Abdullah University of Science and TechnologyThuwal, Saudi Arabia

**Keywords:** *Epulopiscium*, coevolution, surgeonfish, Red Sea, 16S rRNA

## Abstract

*Epulopiscium* is a group of giant bacteria found in high abundance in intestinal tracts of herbivorous surgeonfish. Despite their peculiarly large cell size (can be up to 600 μm), extreme polyploidy (some with over 100,000 genome copies per cell) and viviparity (whereby mother cells produce live offspring), details about their diversity, distribution or their role in the host gut are lacking. Previous studies have highlighted the existence of morphologically distinct *Epulopiscium* cell types (defined as morphotypes A to J) in some surgeonfish genera, but the corresponding genetic diversity and distribution among other surgeonfishes remain mostly unknown. Therefore, we investigated the phylogenetic diversity of *Epulopiscium*, distribution and co-occurrence in multiple hosts. Here, we identified eleven new phylogenetic clades, six of which were also morphologically characterized. Three of these novel clades were phylogenetically and morphologically similar to cigar-shaped type A1 cells, found in a wide range of surgeonfishes including *Acanthurus nigrofuscus*, while three were similar to smaller, rod-shaped type E that has not been phylogenetically classified thus far. Our results also confirmed that biogeography appears to have relatively little influence on *Epulopiscium* diversity, as clades found in the Great Barrier Reef and Hawaii were also recovered from the Red Sea. Although multiple symbiont clades inhabited a given species of host surgeonfish and multiple host species possessed a given symbiont clade, statistical analysis of host and symbiont phylogenies indicated significant cophylogeny, which in turn suggests co-evolutionary relationships. A cluster analysis of *Epulopiscium* sequences from previously published amplicon sequencing dataset revealed a similar pattern, where specific clades were consistently found in high abundance amongst closely related surgeonfishes. Differences in abundance may indicate specialization of clades to certain gut environments reflected by inferred differences in the host diets. Overall, our analysis identified a large phylogenetic diversity of *Epulopiscium* (up to 10% sequence divergence of 16S rRNA genes), which lets us hypothesize that there are multiple species that are spread across guts of different host species.

## Introduction

*Epulopiscium* is an unusual group of enteric symbionts found in high abundance amongst herbivorous surgeonfishes (family: Acanthuridae; Fishelson et al., 1985; Montgomery and Pollak, 1988a; Clements et al., 1989). They were initially identified as protists due to their extremely large cell size (up to ∼600 μm; Fishelson et al., 1985), but close examination of their ultrastructural features suggested them to be prokaryotic (Clements and Bullivant, 1991). Their status as bacteria was then confirmed by 16S rRNA gene analysis, which indicated *Clostridium* species as their closest relatives (Angert et al., 1993), and more specifically the lineage encompassing the cluster XIVb *Clostridia* (Collins et al., 1994). In the absence of isolates, various culture-independent studies shed light on their highly unusual characteristics, such as large cell size, extreme polyploidy (up to 50,000–120,000 copies of single-copy marker genes in mature cells; Mendell et al., 2008), and for some of them, a viviparous reproduction cycle, where live offspring emerge from mother cells (Fishelson et al., 1985; Angert and Clements, 2004; Ward et al., 2009).

Earlier study from the Great Barrier Reef (GBR) noted a high morphological diversity of *Epulopiscium*-like giant bacteria in certain surgeonfishes, and categorized these into 10 ‘morphotypes’ according to their shape, size and reproductive strategy (Clements et al., 1989; summarized in **Table 1**). Briefly, type A were described as largest cigar-shaped cells that can reach up to ∼600 μm and form internal daughter cells; type B cells were more rounded in shape (described as oval-shape) and smaller (<230 μm); types C and D cells were similar in shape to type A, but smaller (40–80 and 8–40 μm, respectively); type E cells were rod-shaped; type F cells were small cigar-shaped cells that seem to reproduce exclusively through multiple phase-bright internal structures (up to 7); type G cells seem to reproduce exclusively by binary fission; type H were heterogeneous cells that contain two distinct internal structures; type I cells were defined by mode of reproduction (binary fission) and the presence of elongate internal structures; and type J were elongate/string-like cells. Note that the phase-bright internal structures described by Clements et al. (1989) were later demonstrated to be endospores by Flint et al. (2005).

**Table 1:** Previously described diversity of *Epulopiscium*.

Fish species	Morphotypes^1^										Location^3^	Methods^4^	Reference
	A^2^	B^2^	C^2^	D	E	F	G	H	I	J^2^			
*Acanthurus auranticavus*	+							+			GBR	Mi	Clements et al., 1989
*Acanthurus bahianus*	Unknown										Atlantic	Ph	Flint, 2006
*Acanthurus blochii*	+						+	+		+	GBR	Mi	Clements et al., 1989
*Acanthurus chirurugus*	Unknown (size: 20–59 μm)										Atlantic	Mi	Grim et al., 2013
*Acanthurus coeruleus*	Unknown (size: 15–60 μm)										Atlantic	Mi	Grim et al., 2013
*Acanthurus dussumieri*	+						+	+			GBR	Mi	Clements et al., 1989
*Acanthurus grammoptilus*	+							+			GBR	Mi	Clements et al., 1989
*Acanthurus lineatus*	+				+			+		+	GBR	Mi	Clements et al., 1989
*Acanthurus mata*	None										GBR	Mi	Clements et al., 1989
*Acanthurus nigricans*	None										GBR	Mi	Clements et al., 1989
*Acanthurus nigricauda*						+		+	+		GBR	Mi	Clements et al., 1989
*Acanthurus nigrofuscus*	++				+		+	+			RS, GBR	Ph	Clements et al., 1989; Angert et al., 1993; Angert, 1995
*Acanthurus nigroris*	+						+	+			GBR	Mi	Clements et al., 1989
*Acanthurus olivaceus*		+				+	+	+			GBR	Mi	Clements et al., 1989; Angert et al., 1996
*Acanthurus pyroferus*							+	+			GBR	Mi	Clements et al., 1989
*Acanthurus thompsoni*	None										GBR	Mi	Clements et al., 1989
*Acanthurus tractus*	Unknown (size: 13–63 μm)										Atlantic	Mi	Grim et al., 2013
*Acanthurus triostegus*	+				+		+	+	+		GBR	Mi	Clements et al., 1989
*Acanthurus xanthopterus*	None										GBR	Mi	Clements et al., 1989
*Ctenochaetus binotatus*						+	+	+			GBR	Mi	Clements et al., 1989
*Ctenochaetus striatus*						+		+			GBR	Mi	Clements et al., 1989
*Ctenochaetus strigosus*						+	+	+			GBR	Mi	Clements et al., 1989
*Naso brevirostris*				+	+		+	+		+	GBR	Mi	Clements et al., 1989
*Naso hexacanthus*	None										GBR	Mi	Clements et al., 1989
*Naso lituratus*			++		+			+		++	GBR, Hawaii	Mi, Ph	Clements et al., 1989; Flint et al., 2005
*Naso tonganus*		+		+	+			+		+	GBR	WGS, Mi, and Ph	Clements et al., 1989; Angert et al., 1993; Angert and Clements, 2004; Miller et al., 2012
*Naso unicornis*			+	+				+		+	GBR	Mi	Clements et al., 1989
*Naso vlamingii*				+	+			+		+	GBR	Mi	Clements et al., 1989
*Zebrasoma scopas*				+	+					+	GBR	Mi	Clements et al., 1989
*Zebrasoma veliferum*				+	+			+		+	GBR	Mi	Clements et al., 1989

^1^*+ denotes presence, ++ denotes two (phylogenetic) subclades within a single morphotype*.

^2^*Bold denote morphotypes with 16S rRNA information*.

^3^*GBR, Great Barrier Reef; RS, Red Sea*.

^4^*Mi = microscopy, Ph = phylogeny inferred by 16S rRNA clone library; WGS, whole genome sequencing*.

However, only four of these morphotypes (A, B, C, and J) have been phylogenetically classified at the 16S rRNA gene level (Angert et al., 1993; Flint et al., 2005), of which three were further split into subclades (A1, A2, C1, C2, J1, and J2) due to large sequence divergences. The large cigar-shaped type A (70–417 μm) had the greatest sequence divergence (>9%), and was separated into two completely different phylogenetic groups (A1 and A2; Angert et al., 1993). Yet, both type A1 and A2 cells found in *Acanthurus nigrofuscus* from geographically disparate locations (the Red Sea and the Pacific) showed a very high 16S rRNA gene sequence similarity (>98%; Angert et al., 1993). Whilst much less divergent, type C — smaller, oval/cigar-shaped and phylogenetically closer to A2 — and type J, the elongate and string-like cells (10–240 μm), were further split into C1, C2, J1, and J2, respectively.

In terms of the distribution, *Epulopiscium* have only been directly observed in herbivorous and detritivorous surgeonfish species from the GBR (**Table 1**; Clements et al., 1989), and this was partially confirmed by the high relative proportions of *Epulopiscium*-like reads (up to 99% in *Naso elegans*) in these fishes in a recent 16S rRNA gene-based pyrosequencing survey conducted in the Red Sea (Miyake et al., 2015). However, some sequences were also recovered from non-herbivorous surgeonfish as well as non-surgeonfishes, albeit at extremely low abundances (<3%); these rare occurrences have been postulated to represent allochthonous populations. Importantly, Clements et al. (1989) also reported association of specific *Epulopiscium* morphotypes with certain host species, in particular related to the host feeding categories. For instance, the type A cells were found exclusively in herbivorous surgeonfishes grazing on hard substrata and mixed substrata, while type F cells were only found in sand-grazing and detritivorous species.

Additionally, host–symbiont cophylogenetic relationships that pertain to coevolution between surgeonfishes and *Epulopiscium* remain unexplored. The life cycle of *Epulopiscium* has been reported to be diurnal and strongly linked to the daily activity of the host fish (Angert and Clements, 2004; Flint et al., 2005), suggesting that the relationship is highly intimate that may support a coevolutionary relationship. To date, direct studies on host and enteric symbiont phylogenies are primarily from plant or invertebrate hosts (Dale and Moran, 2006; Moran et al., 2008). For instance, Noda et al. (2007) found strict phylogenetic associations between termites (Rhinotermitidae), cellulolytic gut symbiont *Pseudotrichonympha* (protists) and intracellular bacterial symbionts of the protists. Similarly, a strong connection between host nematodes and the developmental stages of their gut symbionts, *Xenorhabdus* sp. and *Photorhabdus* sp. (Forst and Nealson, 1966), was reflected by complete congruence in their phylogenies (Maneesakorn et al., 2011). Other examples that are suggestive of coevolutionary relationship between the host and specific gut symbionts have also been reported (e.g., Russell et al., 2009 that explored the evolution of gut symbionts in herbivorous ants). The surgeonfish-*Epulopiscium* symbiotic relationship is likely to be more complex, as multiple symbiont morphotypes were found among multiple host species (Clements et al., 1989).

Thus far, little is understood about the diversity and abundance of *Epulopiscium* in surgeonfishes at the molecular level. In this study we therefore conducted 16S rRNA gene surveys of *Epulopiscium* in several unrelated surgeonfishes from the Red Sea to assess their phylogenetic diversity and distribution among the different hosts. These datasets allowed us to address the following three questions: (1) how extensive is the phylogenetic diversity and the abundance of *Epulopiscium* in different surgeonfishes from the same locale? (2) Do the different *Epulopiscium* morphotypes represent coherent phylogenetic clusters? Lastly, (3) do specific phylogenetic clusters preferentially associate with certain host fishes (cophylogeny), suggesting their coevolution?

## Materials and Methods

### Sample Collection and DNA Extraction

Fish specimens were collected from Al Fahal reef (E38.57935, N22.18344) on the Saudi Arabian coast of the central Red Sea. This research was carried out under the general auspices of King Abdullah University of Science and Technology’s (KAUST) arrangements for marine research with the Saudi Arabian Coast Guard and the Saudi Arabian Presidency of Meteorology and Environment. These are the relevant Saudi Arabian authorities governing all sea-going research actions in the Saudi marine environment. KAUST has negotiated a general and broad permission for marine research in Saudi Arabian Red Sea waters with these two agencies and thus there is no permit number to provide. The study was approved by the Internal Bioethic Committee (IBEC) at KAUST under the permit 15IBEC31_Stingl. All fishes were collected within an hour (8–9 am) to minimize time-dependent effects, and were immediately placed on ice for dissection in the laboratory later on the same day. Three replicates per species of surgeonfishes: *A. nigrofuscus, Acanthurus sohal, Acanthurus gahhm, Ctenochaetus striatus, N. elegans, Naso unicornis, Naso hexacanthus, Zebrasoma desjardinii* and *Zebrasoma xanthurum*, as well as two parrotfish species, *Chlorurus sordidus* and *Scarus niger*, and a rabbitfish species, *Siganus stellatus*, were captured for the analysis. From the same coordinate, coral pieces were collected in a sterile bag, which was later subject to air blowing in the laboratory to collect mucus layer and biofilms, to test for presence of *Epulopiscium*-like bacteria outside the host using *Epulopiscium*-specific PCR primer designed in this study as described below. Once the fishes were transported back to the laboratory, the processing of the fin and gut samples (including DNA extractions) were performed as previously described in Miyake et al. (2015), with the exception that aliquots of all gut samples were also fixed in 4% formaldehyde for microscopy.

The dietary information of the host fishes is provided in Supplementary Table S1. The fishes were categorized into: (i) macroscopic brown algae feeders with high SCFA profiles in the hindgut region; (ii) turfing and filamentous red and green algae feeders with moderate SCFA; (iii) zooplankton feeders with moderate SCFA; (iv) detritus and sedimentary material feeders (detritivores) with low levels of SCFA; and (v) omnivores that feed on both detritus and planktons that seem to show individual differences. Most of the in-depth dietary analysis was conducted in the Indo-Pacific or western Indian Ocean, but was confirmed for the fishes in this study by a brief analysis of stomach contents (Supplementary Table S2). *A. nigrofuscus* was classified as turfing and filamentous red and green algae feeders, although epiphytic diatoms are also consumed seasonally in the Red Sea (Fishelson et al., 1987; Montgomery et al., 1989). *A. sohal* was also classified as turfing and filamentous red and green algae feeders as reported by Vine (1974) and Alwany et al. (2005). *Z. xanthurum* has also been reported to use similar dietary sources to *A. nigrofuscus* and thus classified as turfing and filamentous red and green algae feeder (Montgomery et al., 1989). In contrast, much less work has been conducted on *Z. desjardinii*, but the few papers available confirmed them to share the same diet as *Z. xanthurum* (Alwany, 2008; Cernohorsky et al., 2015), which was corroborated by stomach content analysis (Supplementary Table S1). *Ct. striatus* was classified as detritivore (e.g., Purcell and Bellwood, 1993; Choat et al., 2002, 2004; Crossman et al., 2005; Marshell and Mumby, 2012) although as with *A. nigrofuscus*, seasonal increase in epiphytic diatoms have been reported (Montgomery and Galzin, 1993). *N. elegans* and *N. unicornis* have been reported to be macroscopic brown algae feeders (Choat et al., 2002, 2004; Crossman et al., 2005; Pinault et al., 2013; Cernohorsky et al., 2015), while *N. hexacanthus* has been reported to be zooplankton feeder (e.g., Choat et al., 2002). Lastly, *A. gahhm* has often been referred to as detrital grazer (much like *Ct. striatus*; Afeworki et al., 2013), but was classified as omnivore here as a few have been observed to feed presumably on plankton in the water column (Miyake, personal observations; Choat, personal communications) — much like its sister species *A. xanthopterus*. This has also been confirmed by brief gut content analysis in this study (Supplementary Tables S1 and S2) and in Miyake et al. (2015).

### Host Phylogeny: PCR, DNA Sequencing, and Phylogenetic Analysis

Fish fin DNA was amplified by polymerase-chain reaction (PCR) using four genetic markers: cytochrome c oxidase (CO1), mitochondrial cytochrome b (cytb), 16S (mt16S), and nuclear intron (ETS2) as described by Klanten et al. (2004). Supplementary Table S3 provides details on primers and PCR conditions. Sequencing, downstream analysis and phylogenetic reconstructions were performed according to Miyake et al. (2015), with the exception that the four genetic markers were separately aligned and concatenated to four-gene-per-sequence alignments as recommended by Gadagkar et al. (2005).

### *Epulopiscium*: PCR, Cloning, DNA Sequencing, and Phylogenetic Analysis

Initially, full-length 16S rRNA gene sequences of gut bacterial DNA were PCR-amplified, cloned, and sequenced as described by Ngugi and Stingl (2012) using the universal bacterial primer (EUB, 27F/1492R) on gut bacterial DNA from the sampled fishes (Supplementary Table S3). The cleaned-up PCR products were bi-directionally sequenced (using the original PCR primers) on an Applied Biosystems 3730× l DNA Analyzer at the Bioscience Core Lab of King Abdullah University of Science and Technology (KAUST). Raw sequences were trimmed using SEQUENCHER 4.9 (Gene Codes Corporation) and initially aligned based on SILVA SSUREF database (ver. 119) in MOTHUR, and chimeric (using the UCHIME algorithm; Edgar et al., 2011) as well as identical sequences were removed. The processed sequences were re-aligned and classified online by SINA (ver. 1.2.9) using the SILVA SEED database (Pruesse et al., 2012). From these, sequences classified as *Epulopiscium* were then imported into ARB (ver. 5.1-org-6213; Ludwig et al., 2004) for phylogenetic analysis.

These full-length *Epulopiscium* sequences were then used to design *Epulopiscium*-specific PCR primers in ARB. The designed primer (579uF/1232R) targeted all known *Epulopiscium* sequences (both from this study and from the SILVA database) as well as *Metabacterium polyspora*, spanning the hypervariable regions V4-V7. *In silico* PCR of the primer on SILVA database online (Klindworth et al., 2013) showed 14 hits with no mismatch, and 6 hits with 1 mismatch. Most of these were *Epulopiscium*, but also included a few from closely related *Cellulosilyticum* sequences. The primer did not amplify with *Escherichia coli* DNA, but consistently amplified with the gut DNA that contained *Epulopiscium* in the earlier 16S rRNA clone libraries. The primer was used for PCR, cloning and sequencing of the *Epulopiscium* fraction of the gut microbiota, as described above (Supplementary Table S3). The downstream analysis was conducted as described for the full-length sequences above, except that redundant sequences were removed by UCLUST (Edgar, 2010) with 99% criterion and imported to ARB for phylogenetic analysis.

*Epulopiscium* phylogenetic analysis was conducted as recommended by Peplies et al. (2008), whereby full-length 16S rRNA gene sequences were used to form the backbone of the phylogenetic tree, and populated by shorter 16S rRNA gene sequences derived from *Epulopiscium* group-specific PCR primer designed in this study. Specifically, the maximum likelihood (ML) tree of full-length 16S full-length, together with reference sequences of *Epulopiscium* and closely related *M. polyspora* from the SILVA database (SSU Ref NR 119), were constructed with 1,000 bootstrap replicates. The topology of the tree was confirmed by neighbor joining (NJ, with 1,000 replicates) as well as Bayesian inference methods [BI, in Geneious v8.0.4 (http://www.geneious.com/)]. The *Epulopiscium* partial sequences (derived using the 579uF/1232R primer set) were then added to the full-length 16S phylogenetic tree using the parsimony function in ARB, without allowing change in the overall tree topology, for more robust phylogeny.

The distinct nodes within the tree that showed sequence diversity of ∼3% were clustered together as ‘clades.’ Sequences that did not affiliate with previously described phylotypes (A1, A2, B, C, J1, and J2) were designated as clades RS01-RS11, where RS stands for the Red Sea. Furthermore, the species delimitation plugin (ver.1.03) in Geneious was used to reaffirm different phylogenetic groups (Masters et al., 2011). The tool utilizes bootstrapping/Bayesian posterior probability and Rosenberg’s probability of reciprocal monophyly (*P_AB_*; Rosenberg, 2007) to assess if different phylogenetic groups are statistical supported. *P_AB_* is the probability that species A, represented by *a* sequences in a clade of *a + b* sequences, will be reciprocally monophyletic with the remaining *b* sequences under the null model of random coalescence. Different clades were then tested to observe similarity and difference in their morphology as described below.

### Fluorescence Microscopy Using DAPI and FISH

A combination of 4′,6-diamidino-2-phenylindole (DAPI) stain and an *Epulopiscium*-specific fluorescent *in situ* hybridization (FISH) probe 1432R, designed by Angert et al. (1993) was used to assess the morphological diversity of *Epulopiscium* and to identify the predominant morphotypes in different surgeonfishes. All samples were prepared as described in Angert et al. (1993). Bright-field and fluorescence microscopy were performed using a Zeiss LSM 710 upright confocal microscope. The lengths of DAPI-stained *Epulopiscium* cells in each sample were measured at 10 randomly selected spots on the slide, and differences in cell size between hosts were assessed by Analysis of Variance (ANOVA) and Tukey’s HSD *post hoc* tests. The cell counts recorded ranged from 24 cells in *Ct. striatus* to 275 in *A. sohal*.

Furthermore, clade-specific FISH probes were designed and used to investigate the morphologies of the newly discovered clades in *A. nigrofuscus, A. sohal*, and *Z. desjardinii*. The FISH probes were designed for RS01-RS08, while A2 and B probes were also re-designed to capture all the sequences from each clade. Probes could not be designed for clades RS09 and RS10 due to the relatively unstable nature of their phylogenetic branches and the lack of suitable target matches in their 16S rRNA hypervariable regions. The design, preparation, optimization, and evaluation of the probes, as well as FISH were performed as described by Daims et al. (2005). Briefly, we constructed probes complementary to 16S rRNA sequences that are 15–25 nucleotides in length using the ‘design probe’ function in ARB. All probes captured 100% of the sequences in their targeted group without any mismatches, but at least 1 or 2 mismatches against non-target sequences, depending on the position of the mismatch. For probes that were used together in the same FISH analysis, we ensured that the target location did not overlap (as indicated by *E. coli* position; Supplementary Table S3). Because of the large size of *Epulopiscium* cells, it was easy to distinguish potentially unspecific hybridization of the probe to non-*Epulopiscium* organisms under the microscope. For each probe, different concentrations of formamide were used to obtain maximum hybridization stringency. Additionally, for probes with non-target *Epulopiscium* sequences containing 1 or 2 mismatches, unlabeled competitor probes that matched 100% to these non-targets were used to prevent unspecific hybridization of non-targets (as indicated in Supplementary Table S3). Pure *E. coli* or *Bacillus* culture did not hybridize to any of these probes, nor did they hybridize to any giant *Epulopiscium*-like cells of different morphology. All probes were labeled with fluorescein (Fluo) or cyanine 3 (Cy3) fluorescent dyes at the 5′ end, with both Fluo and Cy3 used for each probe. The optimum FISH protocol as well as further information on hybridisation protocols is described in Supplementary Table S3. Probes for RS04 and RS07 were disregarded from the study as no hybridization was observed, although there remains a possibility that these clades were not present in the prepared samples. For FISH positive clades, cell size was recorded and statistically assessed for significant differences between clades using ANOVA and Tukey’s honest significant difference (HSD) tests.

### Abundance and Distribution Analysis

To robustly assess the relative abundance and distribution of different *Epulopiscium* clades in surgeonfishes, we re-analyzed pyrosequencing data recently published by Miyake et al. (2015), specifically focusing on the *Epulopiscium*-related reads in these datasets. Briefly, the reads from surgeonfishes were aligned and classified based on the SILVA SSURef database (ver. 119), and those identified as *Epulopiscium* were selected (at 80% confidence threshold). These *Epulopiscium* sequences were then reclassified to the different *Epulopiscium* clades identified by full-length 16S rRNA gene phylogeny in this study (see above), using the naïve Bayesian classification method at 80% confidence cut-off (Wang et al., 2007), as implemented in MOTHUR. Sequences that could not be confidently assigned to the clades were labeled as unclassified. The sequence assignment to clades was further validated using Pplacer, which locates reads onto a reference phylogeny by maximizing the phylogenetic likelihood or the posterior probability (Matsen et al., 2010). Clade-specific abundances (for each sample) relative to the total bacterial pyrosequence reads per sample and the clustering of related samples were determined using R (ver. 3.1.3) based on a Bray–Curtis dissimilarity matrix. The resulting matrix was then subjected to hierarchical clustering by complete linkage method and visualized by UPGMA dendogram. The statistical R package, Adonis (McArdle and Anderson, 2001), was then used to test for significant differences in *Epulopiscium* clade abundance between host genera, species and diet (as categorized above) at 1,000 permutations.

### Cophylogeny

Furthermore, we investigated the cophylogenetic relationship between surgeonfishes and *Epulopiscium*. While various methods for studying host–symbiont cophylogeny are well-documented (Stevens, 2004), conventional cost-based methods were impractical for this study because of the complexity of the surgeonfish-*Epulopiscium* relationship, where multiple symbionts were found amongst multiple hosts, making the cost calculation computationally intensive. Importantly, cost-based methods require some understanding on the host–symbiont relationship for deriving assumptions of relative costs of different evolutionary events (e.g., co-speciation, host switching, duplication, and lineage sorting), which are still lacking for this system.

For our purpose, we elected to use two independent global fit models, specifically the Procrustean Approach to Cophylogeny (PACo; Balbuena et al., 2013) and AxParaFit (Legendre et al., 2002; Stamatakis et al., 2007), to statistically assess the host–symbiont cophylogeny. PACo places the host and symbiont phylogenies into separate distance matrices, which are then transformed by principle coordinates. These are then superimposed by Procrustes method using the information on the host–symbiont link. The analysis yields a global goodness-of-fit statistic, whose significance can be established by a randomization procedure. Similarly, AxParaFit is essentially a principle component analysis that uses permutations to calculate the probability of obtaining a null hypothesis. The null hypothesis can be rejected for any interaction with a random tip mapping of 500 iterations. The output of both models indicates the significance of the overall host–symbiont relationship and of interactions between individual links that contribute to the overall pattern. PACo was run in *R-project* (ver. 3.1.1) using the script provided by Balbuena et al. (2013), and AxParaFit was run through CopyCat GUI (Meier-Kolthoff et al., 2007), both with 10^5^ permutations.

### Data Accessibility

Sequences of fish fin genetic markers (CO1, cytb, mt16S, and ETS2) have been submitted to GenBank under accession numbers KT953164 – KT953199 (cytb), KT952598 – KT952633 (mt16S), KT953200 – KT953235 (ETS2). Sequences for CO1 were not submitted due to being identical to previously submitted sequences (KJ658899 – KJ658957). Non-redundant *Epulopiscium* 16S rRNA sequences have been submitted under accession numbers KT952527 – KT952597 for ‘full-length’ and KT952879 – KT953163 for ‘partial’ sequences. Although not used in this study, the representative 16S rRNA sequences of the non-*Epulopiscium* enteric bacteria have also been submitted to GenBank under accession numbers KT952634 – KT952878.

## Results

### Surgeonfish Phylogeny and Diet

**FIGURE 1:**
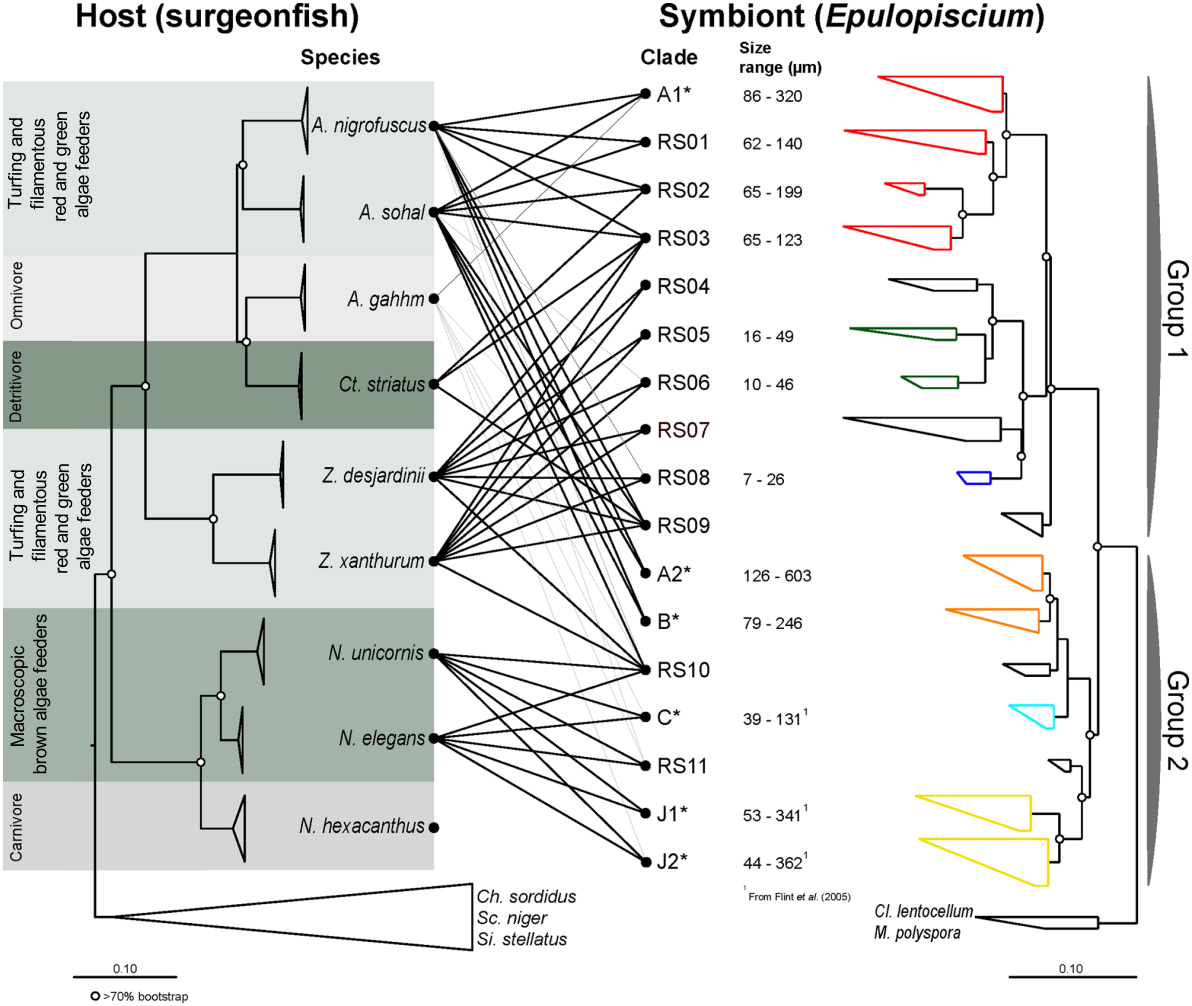
**Maximum-likelihood (ML) phylogenies of surgeonfishes (left) from the central Red Sea and their putative *Epulopiscium*-like symbionts (right) based on trees constructed using multiple marker genes (CO1, CytB, ETS2, and mt16S) and the 16S rRNA gene respectively**. The diet category is as according to Choat et al. (2002, 2004). Lines in the middle represent host–symbiont associations observed, with black lines based on sequences derived from clone libraries (this study) or gray lines based on pyrosequencing data (Miyake et al., 2015). *Epulopiscium* clades that have previously been morphologically characterized elsewhere (in Hawaii or the Great Barrier Reef) are starred (^∗^). Open circles denote branches with bootstrap support values of >70%. The color on the symbiont tree indicates their known morphology: (1) red denotes cigar-shaped clades; (2) green and blue denote short-elongate clades; (3) orange denotes oval/cigar-shaped clades; (4) light blue denotes small cigar-shaped clades; and (5) yellow denotes long-elongate clades.

Nine surgeonfish (*A. gahhm*, *A. nigrofuscus, A. sohal, Ct. striatus, N. elegans, N. hexacanthus, N. unicornis, Z. desjardinii*, and *Z. xanthurum*) and three non-surgeonfish species (*Ch. sordidus, Sc. niger*, and *Si. stellatus*) totaling 36 individuals were collected from the central Red Sea (Supplementary Table S2). We used four marker genes, CO1, CytB, ETS2, and mt16S (aligned to 658, 800, 547, and 599 bases, respectively) to accurately resolve the phylogeny of these fishes. The phylogeny constructed from concatenating these genetic markers was consistent across NJ, ML and BI methods with high bootstrap support (>80%; **Figure 1**). The overall topology was consistent with previous morphological (Winterbottom and Mclennan, 1993) and phylogenetic studies (Klanten et al., 2004; Sorenson et al., 2013). In contrast to our previous work that only used CO1 gene, *A. gahhm* and *Ct. striatus* clustered together within the *Acanthurus* clade because CO1 is relatively conserved amongst surgeonfishes (Miyake et al., 2015).

The stomach content analysis (Supplementary Table S1) was consistent with previous studies, and confirmed that: *N. elegans* and *N. unicornis* are macroscopic brown algae feeders, *A. nigrofuscus, A. sohal, Z. desjardinii*, and *Z. xanthurum* are turfing and filamentous red and green algae feeders, *N. hexacanthus* is a zooplankton feeder, *Ct. striatus* as well as three non-surgeonfishes are detritus and sedimentary material feeders, and *A. gahmm* is an omnivore (Supplementary Table S1). For turfing and filamentous red and green algae feeders, *Acanthurus* species possessed higher proportion of red algae, while *Zebrasoma* species possessed more green algae. Our own brief analysis of the stomach for this study was broadly consistent with their results, except that *A. nigrofuscus* gut contained small amount of sediments (Supplementary Table S2). Unfortunately, one individual each from *A. gahhm*, *A. nigrofuscus*, *N. hexacanthus*, and *Sc. niger* had empty stomachs.

### Morphological Diversity Identified by DAPI and FISH Staining

We used DAPI and FISH [probe 1432R, designed by Angert et al. (1993)] to evaluate the cell size distribution and identify dominant morphotypes of *Epulopiscium* in different species of surgeonfish. We observed no giant *Epulopiscium*-like cells (typically identified as cigar-shaped) or FISH signals in any of the three replicates of *A. gahhm* or *N. hexacanthus*, and from only one of *Ct. striatus* replicates. For other surgeonfishes, the recorded cell sizes and shapes indicated that phylogenetically closely related surgeonfish species were predominated by morphologically similar *Epulopiscium* cells as previously addressed by Clements et al. (1989; Supplementary Figure S1). These cell sizes and shapes were consistent among replicates, although the relative abundances differed. In general, *Epulopiscium* cells in *A. nigrofuscus* and *A. sohal* were significantly larger than those in other surgeonfishes (Tukey’s HSD test on ANOVA, *P* < 0.001; Supplementary Figure S1B). Most of them were cigar-shaped and resembled morphotype A, averaging 112 μm in *A. nigrofuscus* and 189 μm in *A. sohal*, respectively. In both fishes, giant cells showed a bimodal distribution, where two distinct size clusters of cigar-shaped cells (approximately >150 μm and <150 μm) were distinguishable.

In contrast, the guts of *Z. desjardinii* and *Z. xanthurum* had predominantly rod-shaped *Epulopiscium* cells (average size 28 and 31 μm, respectively), similar to type E cells found in *Zebrasoma scopas* (Clements et al., 1989), while the two herbivorous *Naso* species (*N. elegans* and *N. unicornis*) were dominated by small cigar-shaped (51 μm, resembling type C) and elongate/string-like cells (57 μm, resembling type J; Supplementary Figure S1A). In both *Zebrasoma* and *Naso* species described here, as well as in *Ct. striatus*, few small cigar-shaped cells (>25 μm) were also observed.

### *Epulopiscium* Phylogenetic Diversity in the Red Sea

Consistent with the microscopy study above, no *Epulopiscium*-like sequences were recovered from clone libraries of *A. gahhm*, *N. hexacanthus*, or non-surgeonfishes (*Ch. sordidus, Sc. niger*, and *Si. stellatus*), while they were only recovered from *Epulopiscium*-specific clone libraries in *Ct. striatus*. For more comprehensive taxonomic composition of gut microbiota from different fishes refer to Miyake et al. (2015). The extensive phylogenetic tree of full-length representative sequences (at 99% sequence identity cut-off) is shown in Supplementary Figure S2.

**FIGURE 2:**
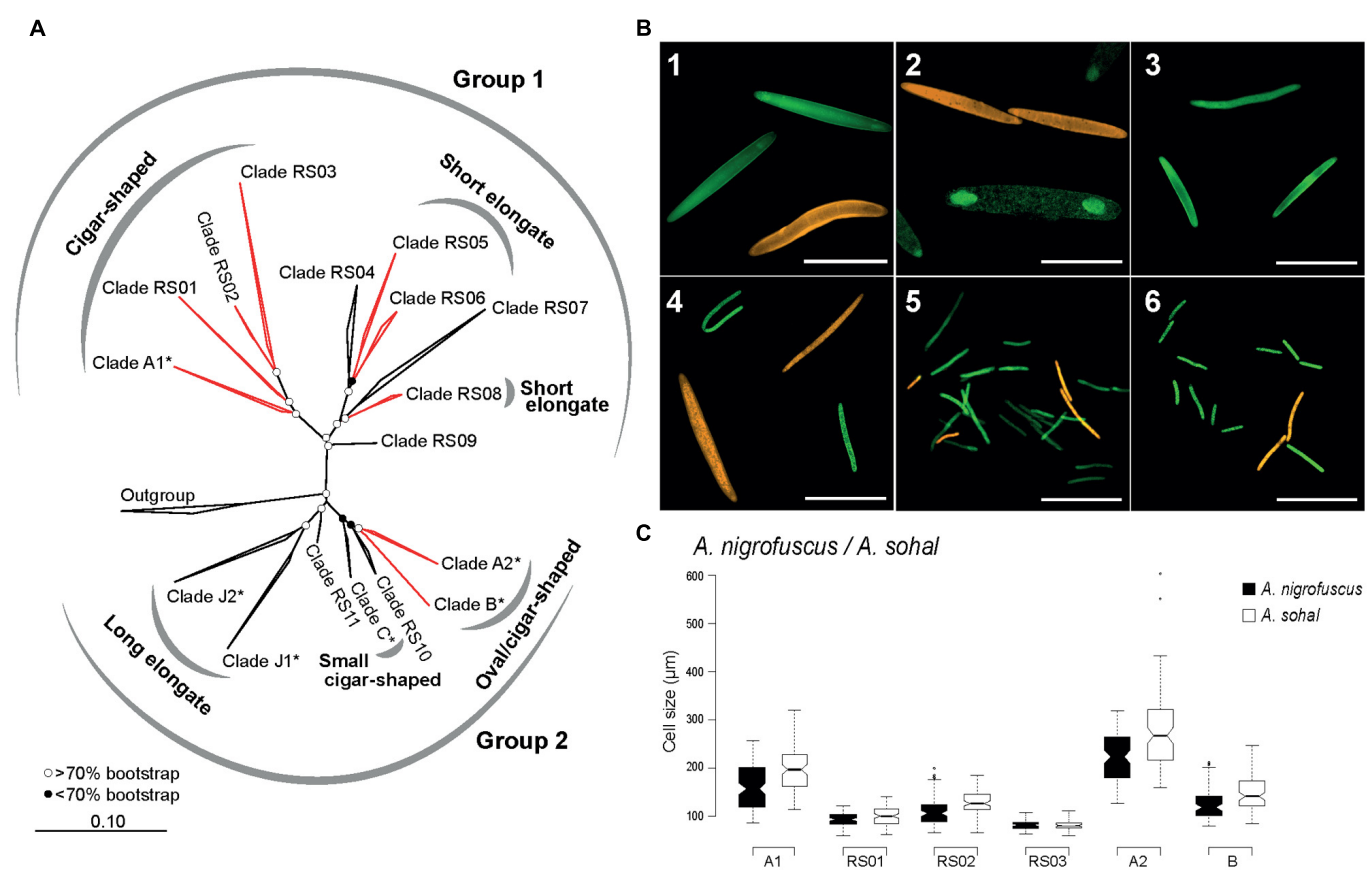
**Phylogenetic and morphological diversity of *Epulopiscium*-like 16S rRNA gene sequences from the central Red Sea**. **(A)** ML 16S rRNA phylogeny. Open and filled circles denote branches with bootstrap support values of >70 or <70%, respectively, based on maximum-likelihood and Bayesian trees. *Epulopiscium* clades that have previously been morphologically characterized elsewhere (in Hawaii or the Great Barrier Reef) are starred (^∗^), and clades investigated by FISH are labeled in red. **(B)** FISH of several clades in *A. sohal* and *Z. desjardinii*: 1) A1 (Cy3-labeled, orange) and A2 (fluorescein-labeled, green) in *A. sohal*, 2) A2 (orange) and B (green) in *A. sohal* – note that A2 cells are internal offspring that still resides within a mother cell, 3) RS01 in *A. sohal* (green), 4) RS02 (orange) and RS03 (green) in *A. sohal* – RS03 cell on the top left corner seems to be undergoing binary fission, 5) RS05 (green) and RS06 (orange) in *Z. desjardinii*, 6) RS06 (orange) and RS08 (green) in *Z. desjardinii*. The scale bars represent 100 μm in images 1–4 and 50 μm in images 5 and 6. **(C)** Cell size distribution of different cigar-shaped clades found in *A. sohal* and *A. nigrofuscus*.

Eighty two full-length (1,434 aligned positions) and 285 partial (680 aligned positions) non-redundant *Epulopiscium*-like 16S rRNA gene sequences were used to construct the phylogenetic trees (**Figures 1** and **2**; also Supplementary Table S4). Overall, the phylogenetic analysis indicated that *Epulopiscium* in surgeonfishes from the central Red Sea were phylogenetically diverse and divergent, with pairwise sequence difference of >10%. Two phylogenetically discrete clusters of *Epulopiscium* sequences – Group 1 and Group 2, were observed in our phylogenetic analyses, which was statistically supported by Rosenberg’s P_AB_ test for reciprocal monophyly (*P_AB_* = 2.5 × 10^-25^) and by high bootstrap values (>70%) for the branch nodes (**Figure 2**). The two groups divided further into 17 distinct monophyletic clades, which had nodes with >70% bootstrap support and a pairwise sequence divergence of ∼3% (Supplementary Table S5). The exceptions were clades RS05/06, RS10, and C, which had bootstrap support <70%, suggesting that the underlying sequences are either highly heterogeneous or are under sampled. Clades previously only identified from other geographically distinct locations (the GBR and Hawaii) were also recovered here (clades A1, A2, B, C, J1, and J2; Supplementary Figure S2). However, in clades where previously published Red Sea sequences were present (A1 and A2; Angert et al., 1993, 1996), the sequences recovered in this study clustered closer to the Red Sea sequences relative to those from the other regions, suggesting an effect of biogeography at sub-clade level. This was not as prominent in A1 where pairwise sequence divergence ranged between 0.8 and 1.6% amongst the Red Sea sequences, but differed by 1.1–1.6% compared to the GBR sequences. However, for A2, the sequences differed by as much as 2.1% between the Red Sea and the GRB, but only 0.4% amongst Red Sea sequences (Supplementary Table S5).

Our study identified eleven new phylogenetic clades (RS01 to RS11) in addition to six that have been previously characterized (i.e., A1, A2, B, C, J1, and J2). Except for sequences within A1, which encompasses previously published sequences, all other clades in Group 2 (RS01 to RS09) essentially represent monophyletic groups of sequences that have not been reported before. Although clade C was previously classified into two clades (C1 and C2; Flint et al., 2005), we did not see a clear separation between C1 and C2 upon inclusion of additional sequences.

### Is *Epulopiscium* Phylogeny Consistent with Morphology?

Given the enormous phylogenetic diversity of *Epulopiscium*, we wanted to analyze the shapes and sizes of the novel clades using newly designed clade-specific FISH probes (Supplementary Table S3) along with those reported in Angert et al. (1993) and Flint et al. (2005). For this purpose, we used gut preparations from *A. nigrofuscus*, *A. sohal* and *Z. desjardinii*, for the simple fact that these fishes harbored novel *Epulopiscium* clades for which probes were designed. Consistent with the earlier phylogenetic data, FISH analyses also showed that clades A1, A2, B, RS01, and RS02 were abundant in *A. nigrofuscus* and *A. sohal* but not in *Z. desjardinii*, while RS05, RS06, and RS08 were found in *Z. desjardinii* but not in *A. nigrofuscus* and *A. sohal* (**Figure 2B**). Contrastingly, RS03 was only detected from the *Acanthurus* hosts (albeit in low abundance), but not in *Zebrasoma*, despite sequences retrieved from all three species. No hybridization was observed for RS07-specific probes from all these fishes.

In terms of morphology, A1 and A2 were large cigar-shaped cells, while most B cells were considerably smaller and were more rounded in shape as previously reported by Angert et al. (1993; **Figure 2B**). However, although A1 and A2 were both cigar-shaped, the former was often not perfectly symmetrical, while the latter was consistently cigar-shaped and laterally symmetrical. Type B cells, which were reported to be ‘oval’ shape — at least in *Naso tonganus* (Ward et al., 2009), were also laterally symmetrical, similar to closely related A2. Additionally, these three clades (A1, A2, and B) were significantly different in size (ANOVA with Tukey’s HSD, *P* < 0.01), with A2 being the largest (126–603 μm) and B the smallest (79–246 μm; **Figure 2C**). Interestingly, there were also host-specific cell size differences, where average sizes were larger in *A. sohal* than in *A. nigrofuscus* for all three clades (average of 161 and 196 μm for A1; 224 and 280 μm for A2 and 124 and 147 μm for B in *A. nigrofuscus* and *A. sohal*, respectively; ANOVA, *P* < 0.01).

Clades RS01, RS02, and RS03 were also cigar-shaped similar to closely related A1, but were significantly smaller in size and less numerous (ANOVA with Tukey’s HSD, *P* < 0.01). Often the cells from these clades were less ‘oval’ in shape than A1/A2/B, but nevertheless were rounded at the polar ends, and thus qualified as ‘cigar-shaped’ for which probes were designed. There was a significant size difference between RS02 (120 μm) and RS03 (84 μm) cells (ANOVA, *P* < 0.01), but this may be due to the lack of adequate sample size, particularly as RS03 was rarely observed. Interestingly, RS03 seems to undergo binary fission (**Figure 2**), which was not observed for RS01 or RS02. Clades A1, RS01, RS02, and RS03 can be grouped together due to having the same morphology and phylogenetic position. This assumption was supported by the high bootstrap value (100%) and significant Rosenberg’s P_AB_ value (*P_AB_* = 9.0 × 10^-12^).

In contrast, cells from the RS05, RS06, and RS08 clades in *Z. desjardinii* were rod-shaped (**Figure 2B**). There was no significant size (or shape) difference between these three clades, although RS05 was the most abundant clade observed. The species delimitation analysis demonstrated that these three clades probably belong to the same group (Rosenberg’s P_AB_ = 4.2 × 10^-4^, bootstrapping = 81%), which is also reflected by their morphological similarity and phylogenetic placement, but the large sequence divergence (>8%) indicates that they are not part of the same ‘species.’ Unfortunately, RS04 and RS07 probe did not hybridize to any cells. Corroboration analyses between morphologies and phylogenies could not be conducted for other clades, due to unstable phylogeny (as was the case for RS09, 10, and 11), or lack of hybridisation (RS04 and RS07).

### Relative Abundance and Distribution of *Epulopiscium*

**FIGURE 3:**
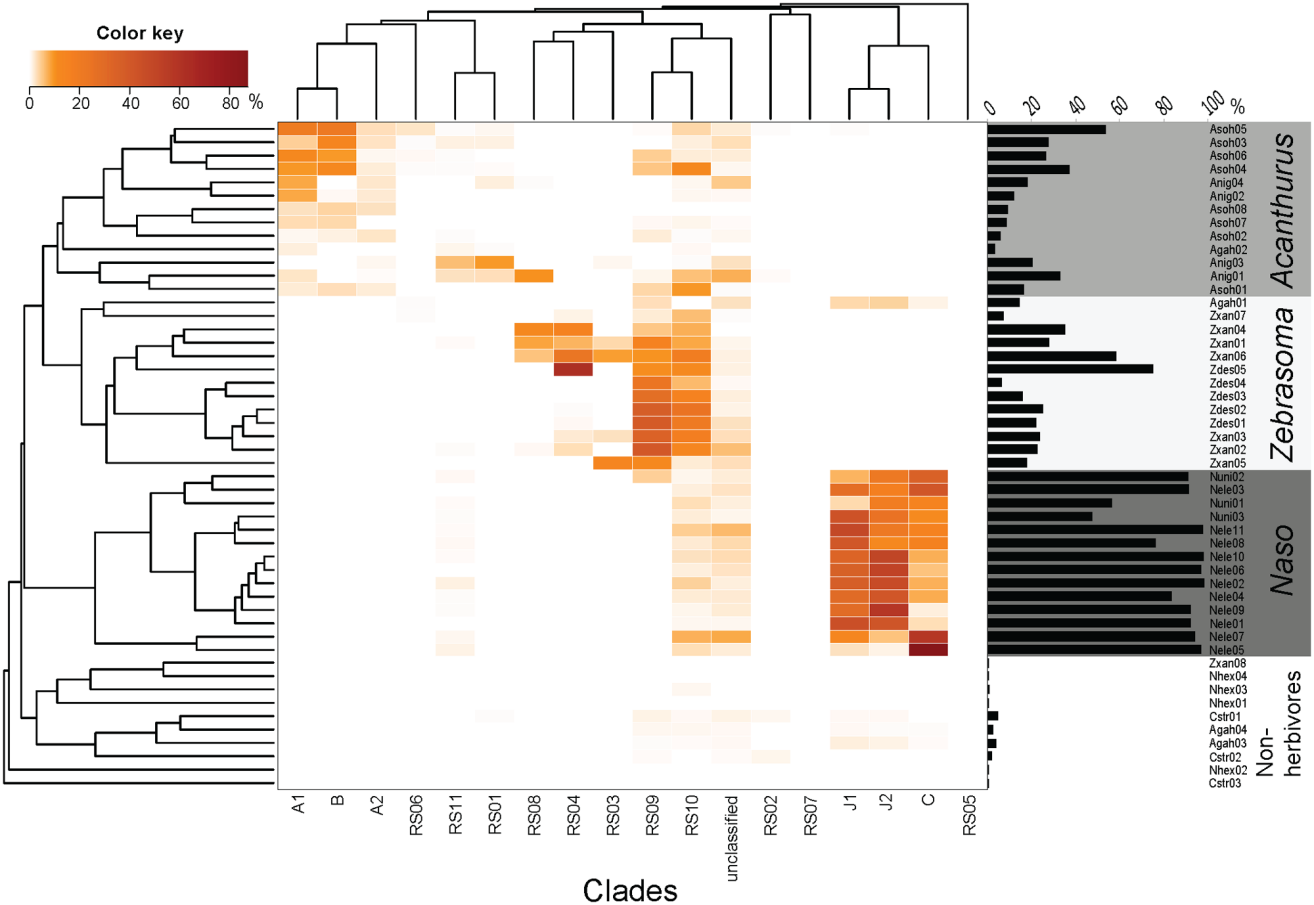
**Relative sequence abundance of different *Epulopiscium* clades from pyrosequencing data of Miyake et al. (2015)**. The Bray–Curtis hierarchical clustering (left) and the *Epulopiscium* relative abundance (right) are also shown. *Epulopiscium* clade abundance formed four main clusters that consisted of herbivorous (*Acanthurus, Zebrasoma*, and *Naso* species) and non-herbivorous surgeonfishes.

Considering the observed phylogenetic and morphological diversity of *Epulopiscium*, we were then prompted to examine in detail the relative abundance and distribution of different clades using a previously published high-throughput 16S rRNA gene dataset in surgeonfishes from different reefs within the central Red Sea (Miyake et al., 2015). A total of 84,550 *Epulopiscium*-like reads (from 178,187 bacterial reads) were retrieved and assigned to the 17 phylogenetic clades (**Figure 3**; Supplementary Table S4). Only about 4.3% of *Epulopiscium*-like reads were not assignable to the phylogenetically defined clades. However, given the high classification threshold that we used (80%), it is likely that most of the *Epulopiscium* diversity within the sampled surgeonfishes was covered in the reconstructed phylogenetic tree. All 17 clades identified from the clone libraries were also found in the amplicon dataset. The pattern of distribution was consistent between the Bayesian and Pplacer classifications, adding further support.

A differential clustering of clade-specific *Epulopiscium* communities was observed in herbivorous hosts, driven by formation of phylogenetically related groupings (Adonis, *P* < 0.001). *Acanthurus* species (*A. nigrofuscus* and *A. sohal*) clustered together due to the dominance of clades A1, A2 and B, whereas *Naso* species (*N. elegans* and *N. unicornis*) groped together principally due to the predominance of C, J1 and J2 clades. Though *Zebrasoma* species (*Z. desjardinii* and *Z. xanthurum*) also grouped together – due to the dominance of clades RS09 and RS10 – many of these clades also occurred in the *Acanthurus* and *Naso* species (e.g., RS05, RS06, RS09, and RS10), albeit at lower abundances. One exception was *Z. xanthurum* sample number 8 (Zxan08) that clustered together with non-herbivorous surgeonfish (**Figure 3**). The non-herbivorous surgeonfishes (*Ct. striatus*, *N. hexacanthus* and two replicates of *A. gahhm*) clustered together probably due to the fact that they possessed low numbers of *Epulopiscium* reads. Some *A. gahhm*, whose *Epulopiscium* abundance varied considerably amongst replicates, clustered with herbivorous *Acanthurus* or *Zebrasoma*. Additionally, the observed clustering was also significantly correlated to the host diet (Adonis, *P* < 0.001).

### Host–Symbiont Cophylogeny

Visualization of *Epulopiscium* clade distribution by Procrustes superimposition plot illustrated that the three main phylogenetic branches of herbivorous (and detritivorous) surgeonfishes — *Acanthurus/Ctenochaetus, Naso* and *Zebrasoma* — possessed distinctive *Epulopiscium* clades (Supplementary Figure S3). Furthermore, the host and symbiont phylogenies were overall statistically significantly correlated (*m*^2^ = 0.31, *P* < 0.001 based on 10^5^ iterations using PACo; Balbuena et al., 2013). The jackknifed-squared residuals were strongest for the species in the *Naso* and *Zebrasoma* genera, which implies stronger association with their resident *Epulopiscium* clades (Supplementary Figure S4). Of 45 interactions, 22 were above the median squared residual value. In particular *Z. xanthurum*-RS03, *A. nigrofuscus*-RS01, *N. elegans*-J2, and *Z. desjardinii*-RS09 showed the largest jackknifed squared residuals. Similarly, AxParaFit (Stamatakis et al., 2007) also confirmed significant global association between the host and symbiont phylogenies (*P* = 0.001, ParaFit Global = 0.004 at 999 permutations). Most of the significant individual links in PACo analysis were also significant with ParaFit Link1 test for individual links, which identifies the individual associations responsible for the global cophylogenetic congruence (those with *P* < 0.05 are shaded in Supplementary Figure S4). However, some interactions below the median squared residual value in PACo analysis were significant with ParaFit Link1 test (e.g., *A. nigrofuscus*-RS03). The strength of congruence increased further when only three most abundant symbiont clades per host were considered (*P* < 0.001).

## Discussion

### *Epulopiscium* Phylogenetic Diversity

Previous studies based on morphological data have indicated that *Epulopiscium* are morphologically diverse in a number of herbivorous surgeonfishes (e.g., Clements et al., 1989), but only a few morphotypes have been phylogenetically classified (Angert, 1995; Flint, 2006). The uncovered phylogenetic diversity of *Epulopiscium*-like bacteria in surgeonfishes from the Red Sea at 16S rRNA gene level varied by as much as 10%, which is well over the threshold used for the genus level taxonomic ranking (Stackerbrandt and Goebel, 1994; Schloss and Handelsman, 2005; Kim et al., 2014). Thus, the two discrete phylogenetic branches encompassing *Epulopiscium fishelsoni* (Angert et al., 1993) and related bacteria, which we confirm here, potentially consist of two genera, each containing multiple species. It remains to be seen whether the novel clades identified in this study are indeed specific to the Red Sea.

No effect of biogeography was evident at clade-level, as similar sequences as those recovered from the GBR and Hawaii (A1, A2, B, C, J1, and J2) were also found in this study. There seems to be some clustering of Red Sea sequences at a subclade level (for instance, A1 sequences from the Red Sea were closer to each other than those from GBR), but this may be caused by the lack of sequences from other regions, and thus would require similar surveys of *Epulopiscium* from reefs around the world to validate. A topographically similar *Epulopiscium* phylogenetic tree has been constructed from the gut of *A. bahianus* in the Atlantic, but the sequences remain unpublished (Flint, 2006). Recently, smaller *Epulopiscium*-like cells, closely resembling the morphotypes F and G, have been described in *A. chirurgus*, *A. coeruleus*, and *A. tractus* from the US Virgin Islands (Grim et al., 2013); however, they have not yet been phylogenetically characterized and it remains to be seen whether they fall within the uncharacterized branches of the phylogeny described here.

Although, not all *Epulopiscium* clades were morphologically characterized, our data suggest that specific morphotypes are also phylogenetically distinct. This was exemplified by cigar-shaped (A1, RS01, RS02, and RS03) and rod-shaped morphotypes (RS05, RS06, and RS08) in this study, as well as string-shaped (type J) and oval-shaped clades from Flint et al. (2005), which were all phylogenetically unique. Rod-shaped clades (RS05, RS06, and RS08) are likely to represent morphotype E, based on descriptions by Clements et al. (1989), while clades RS01-03 seem to represent type D or I [smaller cigar-shaped morphotypes as described by Clements et al. (1989)], but differed by their size range, presence/absence of internal structures or apparent evidence for binary fission.

### *Epulopiscium* Clade Abundances in Different Host Species

*Epulopiscium* clades showed a host-specific abundance pattern amongst herbivorous surgeonfishes which was significantly correlated to the host’s phylogeny and inferred diet. This has also been reported for the community composition of the entire gut microbiota (Miyake et al., 2015), but here we extend this finding to a particular group of highly unusual and abundant symbionts. Relationships amongst host feeding categories and *Epulopiscium* morphotypes have been reported previously (Clements et al., 1989). In light of the high abundance of *Epulopiscium* in most of these herbivorous surgeonfishes (Miyake et al., 2015), the previous clustering patterns observed in the gut microbiota are likely to be influenced by them, with this study further providing the source for that covariance — that is, the differences in *Epulopiscium* clades among the hosts. One exception to the clustering was a single individual of *Z. xanthurum* (Zxan08) that clustered with non-herbivorous surgeonfishes. This is most likely caused by the extremely low abundance of *Epulopiscium* in this sample (∼0.3%). The actual clades found in Zxan08 were similar to those in other *Z. xanthurum* samples (RS03, RS04, RS10, and unclassified), and they in fact clustered together if only clade composition (disregarding the abundance) of clades were considered (data not shown). Either way, this particular individual seems to be an exception to the rule, which was also indicated by a significantly different gut microbiota compared to other *Z. xanthurum* samples (Miyake et al., 2015).

The *Epulopiscium* community within *A. nigrofuscus* clustered to those of phylogenetically related turfing and filamentous algae feeder (*A. sohal*), while the total gut microbiota resembled more closely to that of non-herbivorous fishes (*A. gahhm, Ct. striatus, N. hexacanthus, Ch. sordidus, Sc. niger*, and *Si. stellatus*). These non-herbivorous fishes possessed a high proportion of allochthonous, transient bacteria according to BLAST search of the closest known relatives (Miyake et al., 2015). Similarly, *A. gahhm* showed inconsistencies in the clustering of *Epulopiscium* clades, where one individual clustered with *Acanthurus*, one with *Zebrasoma* and two with non-herbivorous surgeonfishes. This is likely due to the fact that they are omnivorous that feed on a range of diet, which was confirmed by a stomach content analysis (Supplementary Table S1). The role of environmental and host genetic factors in shaping the individuality in the gut microbiota composition has been reported in mice previously (Benson et al., 2010).

Additionally, because closely related hosts have similar nutritional ecology, we cannot conclusively distinguish the relative contribution of the phylogeny (i.e., coevolution) to that of co-occurrence due to the host’s diet in shaping the clustering pattern. However, different clades dominated in *A nigrofuscus*/*A. sohal* compared to *Z. desjardinii*/*Z. xanthurum*, despite all being turfing and filamentous red and green algae feeders indicating that host-dependence in shaping the *Epulopiscium* population. The role of host phylogeny in shaping the gut microbiota as a whole has been explored at subspecies level in zebrafish (Roeselers et al., 2011) and Trinidadian guppies (Sullam et al., 2015), and to species level in surgeonfish (Miyake et al., 2015), while much broader connections have been made for vertebrates (Ley et al., 2008), although host diet is also implicated. Likewise, we argue that in the surgeonfish-*Epulopiscium* symbiosis, both host phylogeny and diet are important factors in shaping the distribution and abundance of *Epulopiscium*. This uncertainty can be resolved by looking at a wider number of surgeonfishes with varying diet.

### The Host–Symbiont Cophylogeny

The phylogenetic data presented here indicated that *Epulopiscium* form complex associations with surgeonfishes, where multiple symbiont clades were found within a given host species and multiple host species harbored a given symbiont clade. The pattern was markedly different to the *sensu stricto* one-to-one host–symbiont associations exemplified by some intracellular symbionts that stems from mother-to-offspring vertical transmission of the symbiont over an evolutionary time scale (e.g., Baumann et al., 1995; Degnan et al., 2004; Hughes et al., 2007; Ikeda-Ohtsubo and Brune, 2009). The fact that *Epulopiscium* has been found in genus *Naso*, the most extant genus of surgeonfish, suggest that the host–symbiont association could be as old as ∼50 million years ago when surgeonfish as a family started to diversify (Sorenson et al., 2013). The direct transfer of symbiont between host mother-offspring has not been observed in *Epulopiscium*, nor any parental care of the larvae has been reported, leaving little room for vertical transmission. Instead, they are likely to be acquired *post partum* from the environment, yet certain clades consistently dominate in the specific host species. They have however, neither been detected in the surrounding seawater (Ngugi et al., 2012; Moitinho-Silva et al., 2013; Thompson et al., 2013), nor in coral mucus and biofilm (Roder et al., 2014, 2015; this study). One possible hypothesis is that transmission might occur by coprophagy, in which organisms consume the feces (Frankenberg and Smith, 1967). Interestingly, coprophagy is reported as the mode of transmission the guinea pig symbiont, *M. polyspora* — the deepest phylogenetic sister group of *Epulopiscium* (Angert et al., 1996). Indeed, coprophagy has been reported in certain species of adult and juvenile surgeonfishes (e.g., *A. nigrofuscus* and *Z. scorpas*; Bailey and Robertson, 1982; Clements, 1997). As noted previously, there remains a possibility that the displayed cophylogeny may be due to an effect of host diet. Elsewhere in vertebrates, the Gram-positive enteric bacterium *Lactobacillus reuteri* has been studied extensively due to its beneficial attributes in human guts (Walter et al., 2011). It is autochthonous in wide variety of hosts, and studies have shown the genomic basis for host specialization (Frese et al., 2011). The phylogenetic analysis of *L. reuteri* from six different host species (human, mouse, rat, pig, chicken, and turkey) clearly reflected the host origin, illustrating a host-driven coevolution of the symbiont (Oh et al., 2010).

### The Importance of Different *Epulopiscium* Clades

The significant cophylogenetic relationship and clade-specific distribution pattern of *Epulopiscium* clades in the different hosts highlight the importance of *Epulopiscium* in herbivorous surgeonfishes, although previous report has indicated that they are able to survive without the symbiont, at least for a few days (Montgomery and Pollak, 1988b). Nevertheless, the drop in enteric pH has been associated with the presence of *Epulopiscium* in *A. nigrofuscus*, which implies that they do indeed play a role in the host gastrointestinal regulation (Pollak and Montgomery, 1994).

One hypothesis for the huge diversity yet host-specific distribution of *Epulopiscium* in surgeonfishes is that the different clusters are specialized for the specific gut condition. For instance, A1, A2, and B may be especially adapted for uptake/breakdown and degradation of different polysaccharides or nutrients resulting from the host specializing in turfing and filamentous algae, while C, J1, and J2 may be better adapted for macroalgae feeders. The diversification of the symbionts due to host specialization has been reported in other systems, particularly in the enteric symbiont *L. reuteri* of mammalian hosts (Oh et al., 2010; Frese et al., 2011). However, the studied hosts were phylogenetically distant (rodents and humans), and there was no clear morphological differentiation in these symbionts. In addition, various enzymes (beta-galactosidase, beta-glucosidase, mannanase, pullulanase/neopullulanase, and xylobiase) responsible for the breakdown of carbohydrates and complex hemicellulose have been found in the published draft genome of morphotype B (Miller et al., 2011), while the closely related *Cellulosilyticum lentocellum* is also known to be cellulose degrading (Miller et al., 2011). However, this does not necessarily say that *Epulopiscium* themselves are directly responsible for the breakdown of algal matter, but rather, they are likely to be one of a series of players that together form a consortia to breakdown the food consumed by the host (Flint et al., 2008). Indeed, such complex microbial system has been studied extensively in termites (e.g., Warnecke et al., 2007; Brune, 2014), while host-specific gut microbiota has been reported for the Red Sea surgeonfishes (Miyake et al., 2015). By adapting their abilities to digest certain types of macronutrients available in specific host gut, *Epulopiscium* might have evolved into diverse clades to exploit different niche environments. To better understand the metabolic diversity of the clades, a better understanding of the constituents in the food resources of different surgeonfishes (e.g., carbohydrate composition) and *in situ* metabolite profiles in their guts, together with a detailed analysis of the genetic repertoires of different *Epulopiscium* clades would be pivotal.

## Conclusion

This study revealed the phylogenetic diversity and distribution of *Epulopiscium* in surgeonfishes from the Red Sea. Eleven previously unexplored clades of *Epulopiscium* were identified and characterized, with multiple ‘species’ apparently existing within multiple ‘genera’ of *Epulopiscium*. Surgeonfish and *Epulopiscium* are intricately associated, as evident by clear dominance of specific clades in a given host species, as well as significant cophylogeny. The clades are likely adapted to specific conditions in the host gut, presumably driven by the diet. Given that various enzymes responsible for the breakdown of carbohydrates and complex hemicellulose have been found in the draft genome (Miller et al., 2012), we speculate further that specific clades found in high abundance may be adapted to the specific gut environment of the host. The study highlighted the importance of *Epulopiscium* to herbivorous surgeonfishes and their host-specific clade specialization, but further investigation into the functional repertoires of different *Epulopiscium* will be essential to unravel their role in the host gut.

## Author Contributions

SM helped to design the study, conducted the experiments and wrote the draft. DN was involved in data analyses and edited the manuscript. US designed the project, and edited the manuscript.

## Conflict of Interest Statement

The authors declare that the research was conducted in the absence of any commercial or financial relationships that could be construed as a potential conflict of interest.
