# Supplementary material for: Phylogenetic Diversity, Distribution, and Cophylogeny of Giant Bacteria (Epulopiscium) with their Surgeonfish Hosts in the Red Sea
Source: Front Microbiol. 2016 Mar 14;7:285. doi: 10.3389/fmicb.2016.00285 (PMC4789555; doi:10.3389/fmicb.2016.00285)
Supplement: Supplementary file 2 [file Image_1.PDF]

Figure S1: Different *Epulopiscium* morphotypes present amongst different surgeonfishes in the Red Sea. **A)** DAPI-stained confocal images of *Epulopiscium*-like giant bacteria from: **1)** *A. nigrofuscus*, **2) & 3)** *A. sohal*, **4)** *N. elegans*, **5) & 6)** *N. unicornis*, **7)** *Z. desjardinii*, **8).** *Z. xanthurum* and **9)** *Ct. striatus*. Note the similarity in the morphology of the cells between closely related hosts. **B)** Size distribution of *Epulopiscium*-like giant bacteria from different host species.

Figure S2: Comprehensive *Epulopiscium* 16S rRNA phylogeny inferred by ML, BI and NJ methods with 1,000 bootstrap replicates. The tree here only shows non-redundant, full-length sequences. Number of partial sequences that clustered within each clades are also shown on the right. Previously published sequences are labelled in red.

Figure S3: Procrustes superimposition plot of surgeonfishes and *Epulopiscium.* The ordinations are based on principal correspondence coordinates of patristic distances. The *Epulopiscium* configuration (dots) has been rotated and scaled to fit the surgeonfish ordination (arrow tips). The length of the arrows represents the projection of the residuals onto the first two axes.

Figure S4: Contributions by the individual surgeonfish-*Epulopiscium* associations to the Procrustean fit. Jack-knifed squared residuals (bars) and upper 95% confidence intervals (error bars) resulted from applying PACo to patristic distances. Bars of associations with significant results in ParaFitLink1 analysis are filled. The red vertical dotted line indicates the median squared residual value.

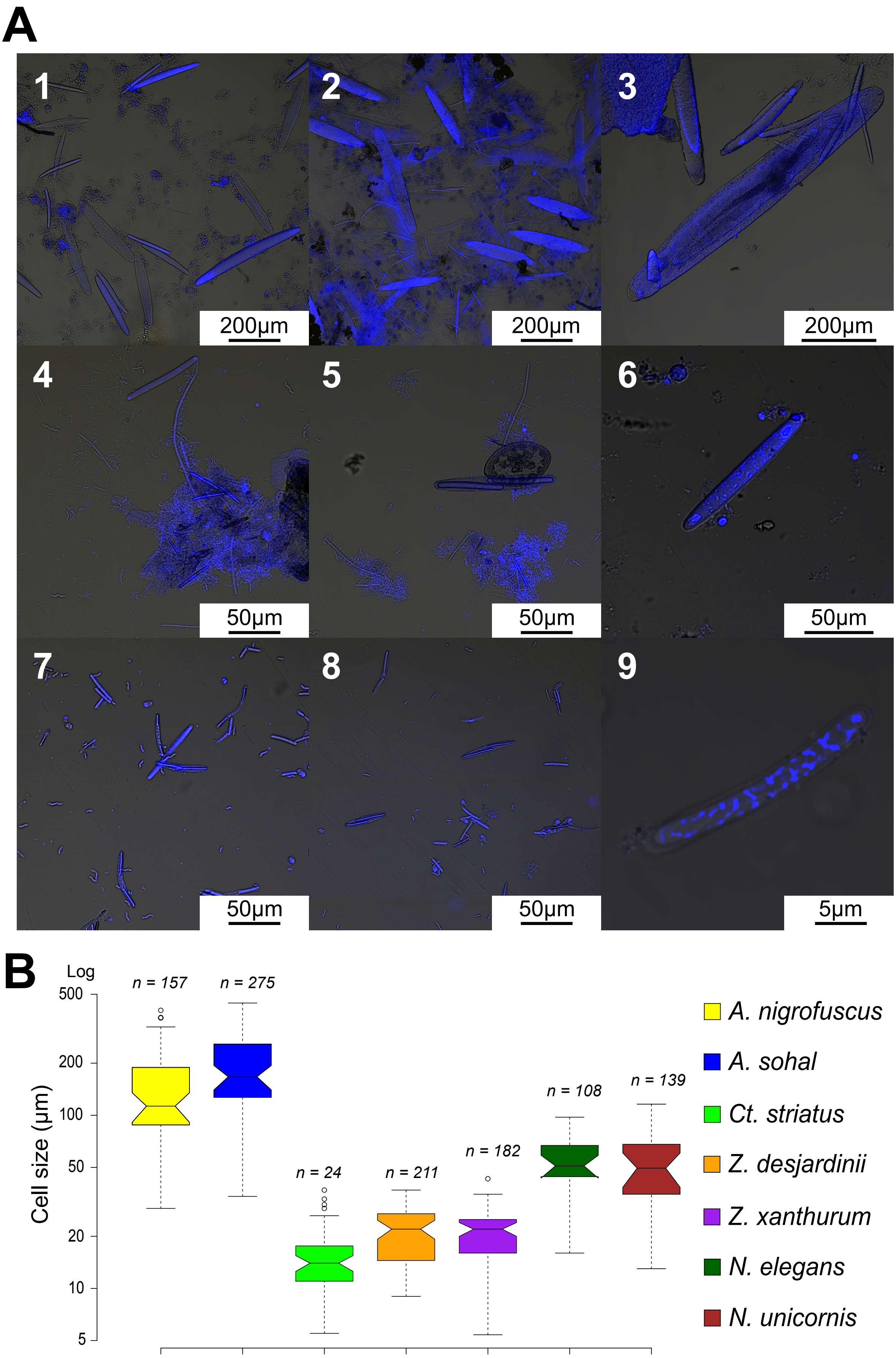

A
1
2
3
200µm
200µm
200µm
4
5
6
50µm
50µm
50µm
7
8
9
50µm
50µm
5µm
B
Log
500
200
100
50
20
10
5
Cell size (µm)
n = 157
n = 275
n = 24
n = 211
n = 182
n = 108
n = 139
A. nigrofuscus
A. sohal
Ct. striatus
Z. desjardinii
Z. xanthurum
N. elegans
N. unicornis

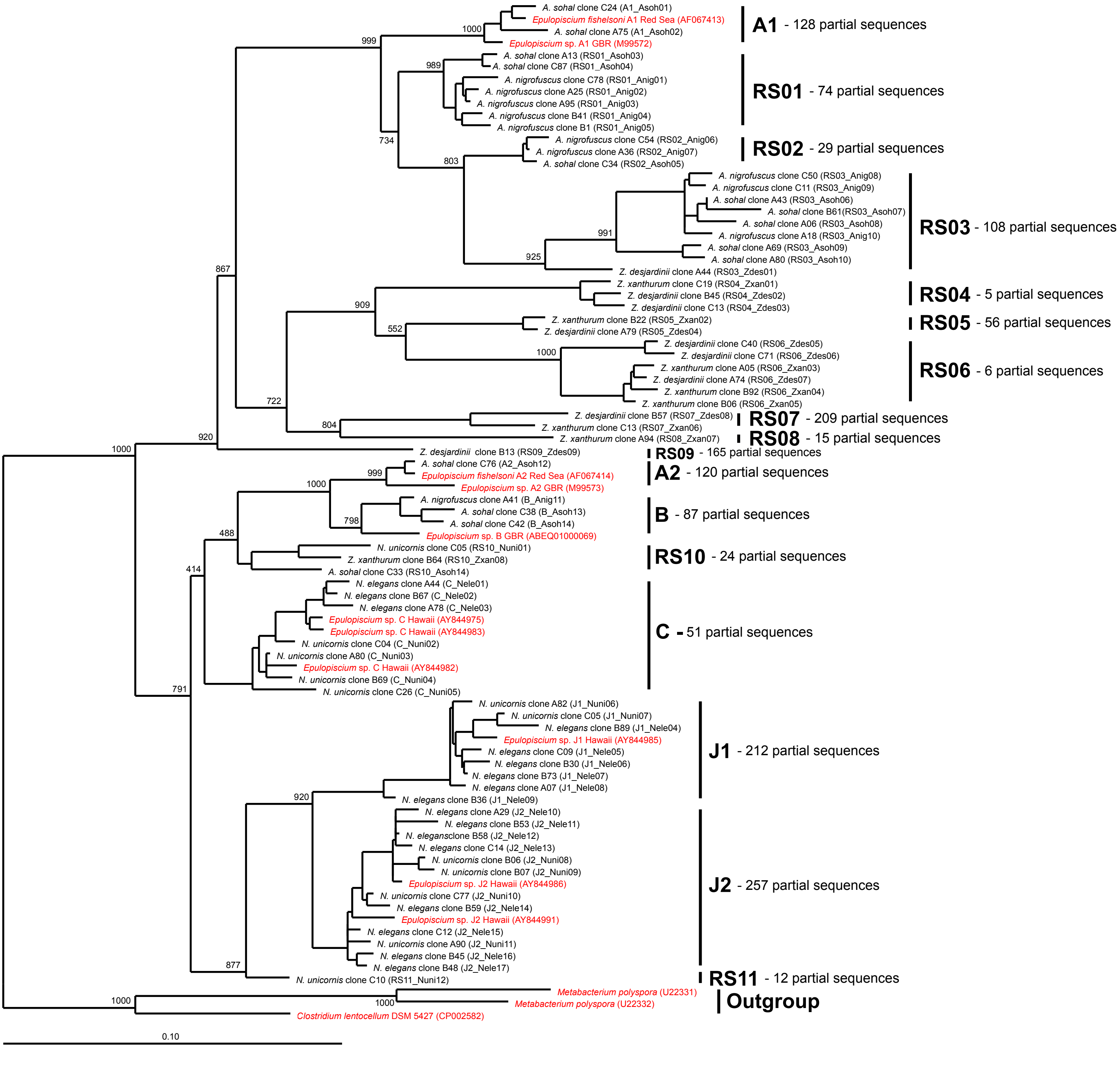
A. sohal clone C24 (A1_Asoh01)
Epulopiscium fishelsoni A1 Red Sea (AF067413)
A. sohal clone A75 (A1_Asoh02)
Epulopiscium sp. A1 GBR (M99572)
A. sohal clone A13 (RS01_Asoh03)
A. sohal clone C87 (RS01_Asoh04)
A. nigrofuscus clone C78 (RS01_Anig01)
A. nigrofuscus clone A25 (RS01_Anig02)
A. nigrofuscus clone A95 (RS01_Anig03)
A. nigrofuscus clone B41 (RS01_Anig04)
A. nigrofuscus clone B1 (RS01_Anig05)
A. nigrofuscus clone C54 (RS02_Anig06)
A. nigrofuscus clone A36 (RS02_Anig07)
A. sohal clone C34 (RS02_Asoh05)
A. nigrofuscus clone C50 (RS03_Anig08)
A. nigrofuscus clone C11 (RS03_Anig09)
A. sohal clone A43 (RS03_Asoh06)
A. sohal clone B61(RS03_Asoh07)
A. sohal clone A06 (RS03_Asoh08)
A. nigrofuscus clone A18 (RS03_Anig10)
A. sohal clone A69 (RS03_Asoh09)
A. sohal clone A80 (RS03_Asoh10)
Z. desjardinii clone A44 (RS03_Zdes01)
Z. xanthurum clone C19 (RS04_Zxan01)
Z. desjardinii clone B45 (RS04_Zdes02)
Z. desjardinii clone C13 (RS04_Zdes03)
Z. xanthurum clone B22 (RS05_Zxan02)
Z. desjardinii clone A79 (RS05_Zdes04)
Z. desjardinii clone C40 (RS06_Zdes05)
Z. desjardinii clone C71 (RS06_Zdes06)
Z. xanthurum clone A05 (RS06_Zxan03)
Z. desjardinii clone A74 (RS06_Zdes07)
Z. xanthurum clone B92 (RS06_Zxan04)
Z. xanthurum clone B06 (RS06_Zxan05)
Z. desjardinii clone B57 (RS07_Zdes08)
Z. xanthurum clone C13 (RS07_Zxan06)
Z. xanthurum clone A94 (RS08_Zxan07)
Z. desjardinii clone B13 (RS09_Zdes09)
A. sohal clone C76 (A2_Asoh12)
Epulopiscium fishelsoni A2 Red Sea (AF067414)
Epulopiscium sp. A2 GBR (M99573)
A. nigrofuscus clone A41 (B_Anig11)
A. sohal clone C38 (B_Asoh13)
A. sohal clone C42 (B_Asoh14)
Epulopiscium sp. B GBR (ABEQ01000069)
N. unicornis clone C05 (RS10_Nuni01)
Z. xanthurum clone B64 (RS10_Zxan08)
A. sohal clone C33 (RS10_Asoh14)
N. elegans clone A44 (C_Nele01)
N. elegans clone B67 (C_Nele02)
N. elegans clone A78 (C_Nele03)
Epulopiscium sp. C Hawaii (AY844975)
Epulopiscium sp. C Hawaii (AY844983)
N. unicornis clone C04 (C_Nuni02)
N. unicornis clone A80 (C_Nuni03)
Epulopiscium sp. C Hawaii (AY844982)
N. unicornis clone B69 (C_Nuni04)
N. unicornis clone C26 (C_Nuni05)
N. unicornis clone A82 (J1_Nuni06)
N. unicornis clone C05 (J1_Nuni07)
N. elegans clone B89 (J1_Nele04)
Epulopiscium sp. J1 Hawaii (AY844985)
N. elegans clone C09 (J1_Nele05)
N. elegans clone B30 (J1_Nele06)
N. elegans clone B73 (J1_Nele07)
N. elegans clone A07 (J1_Nele08)
N. elegans clone B36 (J1_Nele09)
N. elegans clone A29 (J2_Nele10)
N. elegans clone B53 (J2_Nele11)
N. eleganclone B58 (J2_Nele12)
N. elegans clone C14 (J2_Nele13)
N. unicornis clone B06 (J2_Nuni08)
N. unicornis clone B07 (J2_Nuni09)
Epulopiscium sp. J2 Hawaii (AY844986)
N. unicornis clone C77 (J2_Nuni10)
N. elegans clone B59 (J2_Nele14)
Epulopiscium sp. J2 Hawaii (AY844991)
N. elegans clone C12 (J2_Nele15)
N. unicornis clone A90 (J2_Nuni11)
N. elegans clone B45 (J2_Nele16)
N. elegans clone B48 (J2_Nele17)
N. unicornis clone C10 (RS11_Nuni12)
Metabacterium polyspora (U22331)
Metabacterium polyspora (U22332)
Clostridium lentocellum DSM 5427 (CP002582)
A1 - 128 partial sequences
RS01 - 74 partial sequences
RS02 - 29 partial sequences
RS03 - 108 partial sequences
RS04 - 5 partial sequences
RS05 - 56 partial sequences
RS06 - 6 partial sequences
RS07 - 209 partial sequences
RS08 - 15 partial sequences
RS09 - 165 partial sequences
A2 - 120 partial sequences
B - 87 partial sequences
RS10 - 24 partial sequences
C - 51 partial sequences
J1 - 212 partial sequences
J2 - 257 partial sequences
RS11 - 12 partial sequences
Outgroup
1000
999
989
734
803
991
925
867
909
552
1000
722
804
920
1000
999
1000
798
488
414
791
920
877
1000
1000
0.10

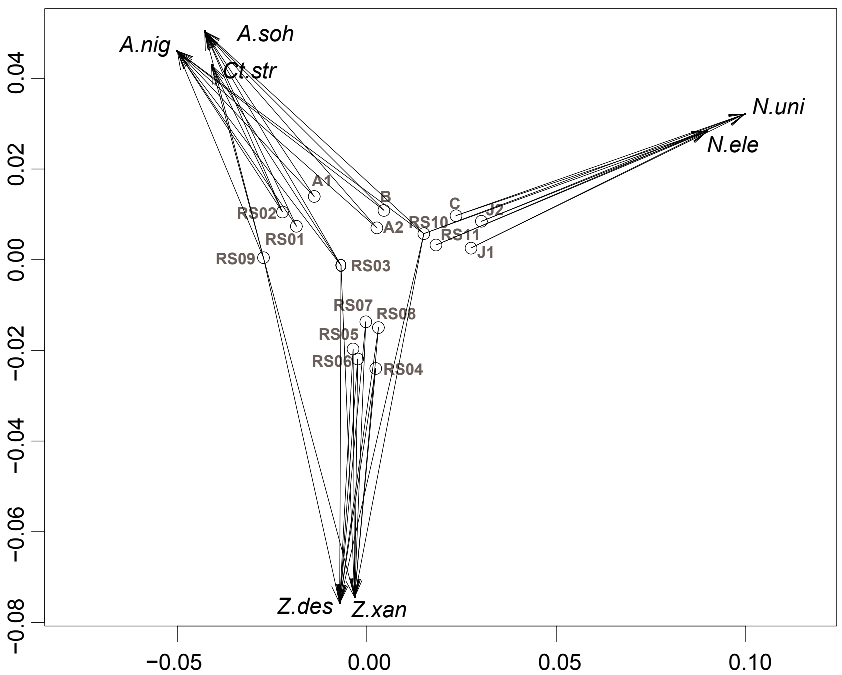

A.nig
A.soh
Ct.str
N.uni
N.ele
Z.des
Z.xan
A1
B
C
J2
J1
A2
RS10
RS11
RS02
RS01
RS09
RS03
RS07
RS08
RS05
RS06
RS04
0.04
0.02
0.00
−0.02
−0.04
−0.06
−0.08
−0.05
0.00
0.05
0.10

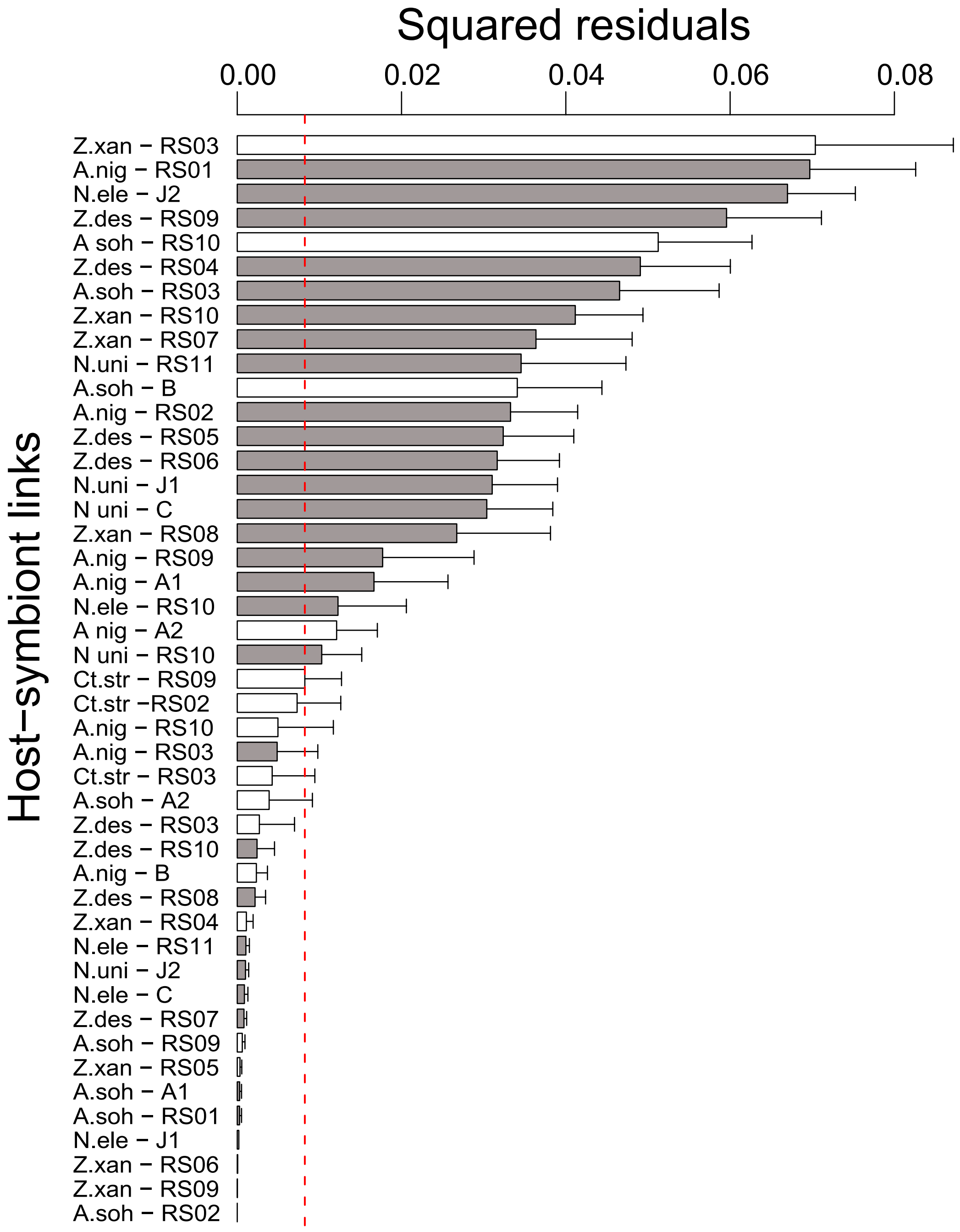
Squared residuals
0.00
0.02
0.04
0.06
0.08
Host−symbiont links
Z.xan − RS03
A.nig − RS01
N.ele − J2
Z.des − RS09
A soh − RS10
Z.des − RS04
A.soh − RS03
Z.xan − RS10
Z.xan − RS07
N.uni − RS11
A.soh − B
A.nig − RS02
Z.des − RS05
Z.des − RS06
N.uni − J1
N uni − C
Z.xan − RS08
A.nig − RS09
A.nig − A1
N.ele − RS10
A nig − A2
N uni − RS10
Ct.str − RS09
Ct.str −RS02
A.nig − RS10
A.nig − RS03
Ct.str − RS03
A.soh − A2
Z.des − RS03
Z.des − RS10
A.nig − B
Z.des − RS08
Z.xan − RS04
N.ele − RS11
N.uni − J2
N.ele − C
Z.des − RS07
A.soh − RS09
Z.xan − RS05
A.soh − A1
A.soh − RS01
N.ele − J1
Z.xan − RS06
Z.xan − RS09
A.soh − RS02
